# The Role of Astrocytes in Multiple Sclerosis

**DOI:** 10.3389/fimmu.2018.00217

**Published:** 2018-02-19

**Authors:** Gerald Ponath, Calvin Park, David Pitt

**Affiliations:** ^1^Department of Neurology, Yale School of Medicine, New Haven, CT, United States

**Keywords:** astrocytes, multiple sclerosis, neuroinflammation, NF-κB, risk variant, leukocyte recruitment

## Abstract

The role traditionally assigned to astrocytes in the pathogenesis of multiple sclerosis (MS) lesions has been the formation of the glial scar once inflammation has subsided. Astrocytes are now recognized to be early and highly active players during lesion formation and key for providing peripheral immune cells access to the central nervous system. Here, we review the role of astrocytes in the formation and evolution of MS lesions, including the recently described functional polarization of astrocytes, discuss prototypical pathways for astrocyte activation, and summarize mechanisms by which MS treatments affect astrocyte function.

## Introduction

Multiple sclerosis (MS) is an autoimmune disease that targets the central nervous system (CNS) (1). It is the most common, non-traumatic neurological disorder in young patients and affects nearly 1 million people in the US alone (2). In the majority of MS patients, the disease manifests itself as episodes of neurological dysfunction that remit spontaneously [relapsing–remitting MS (RRMS)] (1). Pathologically, relapses are associated with focal, inflammatory demyelination in white and gray matter, characterized by infiltration with macrophages and T and B lymphocytes (3). Over two-thirds of patients eventually develop secondary progressive MS (SPMS), a disease stage that is believed to be driven by neurodegeneration, where patients experience slow and irreversible accumulation of disability, predominantly affecting ambulation and cognition (1, 4). In a small percentage of MS patients, progression sets in at disease onset, a disease course that is termed primary progressive MS (1). The pathophysiology of primary and secondary progression remains largely unexplained; however, multiple lines of evidence suggest that progressive MS is associated with chronic activation of the CNS innate immune system (5–7). The poor understanding of the pathomechanisms underlying progression is reflected in the current treatment options for MS, with 13 FDA-approved medications being available for RRMS, one moderately effective medication for primary progressive MS, and none for secondary progression (8).

Multiple sclerosis is the result of an interplay between environmental and genetic factors. Genome-wide association studies (GWASs) have identified over 230 variants associated with susceptibility for MS that all confer small increases in disease risk (9–11). Environmental factors associated with MS risk include smoking, childhood obesity, low vitamin D levels, infection with the Epstein–Barr virus, and possibly a high salt diet (12–14). The pathological hallmark of MS is the presence of focal inflammatory lesions characterized by primary demyelination and relative preservation of axons (15). Acute demyelinating lesions are populated by abundant foamy, myelin-laden macrophages and by lymphocytes that are located in the perivascular space and diffusely throughout the lesion area, albeit at much lower numbers than myeloid cells (15). Acutely demyelinating lesions eventually evolve into chronic active lesions, which are characterized by completed demyelination and astroglial scarring in the lesion center, and inflammatory cells at the lesion rim, possibly associated with low-grade demyelination (15). Finally, chronic silent lesions consist of astroglial scars with few or no inflammatory cells (15). Astrocytes have traditionally been assigned a bystander role, reacting only once demyelination is completed by forming a glial scar (16). However, recent literature suggests that astrocytes are early and highly active participants in MS lesion development (17–19). Astrocytes play multiple roles in the evolution of MS lesions, not only by recruiting lymphocytes (19, 20) and contributing to tissue damage (21–24) but also by confining inflammation and promoting lesion repair (18). In addition, astrocytes themselves sustain significant damage during the inflammatory process (16). This review focuses on the contributions of astrocytes to MS lesion formation. We discuss astrocytic phenotypes, prototypical pathways for astrocyte activation, including the impact of genetic risk variants for MS susceptibility on astrocyte responses, and mechanisms by which MS treatments affect astrocyte function.

## The Physiological Role of Astrocytes and Astrocyte Responses

Astrocytes make up approximately 30% of glial cells in the CNS, where each astrocyte occupies a unique territory demarcated by non-overlapping, star-shaped processes that extend from the cell soma (25, 26). The distal end feet of these processes form the glia limitans when they envelop the parenchymal basal lamina associated with blood vessels or meninges (18, 25). The glia limitans contributes to the maintenance of blood–brain barrier (BBB) integrity and forms a secondary barrier that further restricts entry of peripheral immune cells into the CNS (16, 27). Astrocytes are paramount for normal CNS functions, including maintenance of glutamate, extracellular potassium, and water homeostasis (20, 25). Astrocytes are functionally connected to adjacent astrocytes and to oligodendrocytes by gap junctions, thereby forming large syncytium-like glial networks that are composed of hundreds of cells (28). Together with neuronal synapses, astrocyte processes form so-called tripartite synapses, where one single astrocyte connects with tens of thousands of neuronal synapses (29) to regulate neuronal synaptic transmission, e.g., by releasing glutamate, d-serine, and ATP (30, 31). Astrocytes also prune synapses through phagocytosis (32) and modify gene expression, e.g., associated with neural plasticity, in surrounding neurons by secreting miRNA-containing exosomes (33). In addition, astrocytes secrete neurotrophic factors (34) and are metabolically coupled to neurons, releasing lactate for neuronal uptake and providing antioxidants such as glutathione and thioredoxin (35, 36). Astrocytes also participate in the production of neurosteroids, such as allopregnanolone, estrogen, and dehydroepiandrosterone (DHEA), that are synthesized in the nervous system, where they modulate neuronal excitability, promote myelination, and dampen pro-inflammatory responses in astrocytes (37–41). Moreover, in the healthy CNS, astrocytes contribute to an anti-inflammatory environment through constitutive low-level secretion of the anti-inflammatory cytokines TGF-β (42) and IL-10 (43), expression of Fas ligand (44, 45), and induction of upregulation of the co-inhibitory cell surface receptor CTLA-4 on helper T cells (46).

Astrocyte reactivity in adaptive and innate immune responses can be triggered through oxidative or chemical stress, pro-inflammatory cytokines, damage-associated molecular patterns (DAMPs), released in the context of CNS tissue damage, and pathogen-associated molecular patterns (PAMPs), such as double-stranded RNA and bacterial membranous endotoxins, released from pathogens (19, 47, 48). Stimulation of astrocytes induces or upregulates astrocytic secretion of cytokines, such as TNF-α, IL-1β, and IL-6; neurotrophic factors including nerve growth factor (NGF), brain-derived neurotrophic factor (BDNF), vascular endothelial growth factor (VEGF), and leukemia inhibitory factor (LIF) (19, 48–50); chemokines including CCL2, CCL20, and CXCL10; and β-defensins, antimicrobial peptides that can directly diminish the stability of bacterial membranes and stimulate various immune functions (51, 52). In addition, reactive astrocytes express cell adhesion molecules such as ICAM-1 and VCAM-1 (50), inducible nitric oxide synthase (iNOS) with concomitant production of reactive nitrogen species (53, 54), and the PAMP-recognizing toll-like receptor 3 (TLR3), while other TLRs remain low to undetectable (55–58). Activation of TLR3 triggers a predominantly neuroprotective response, characterized by secretion of growth and differentiation mediators as well as pro- and anti-inflammatory cytokines (58).

**Figure 1:**
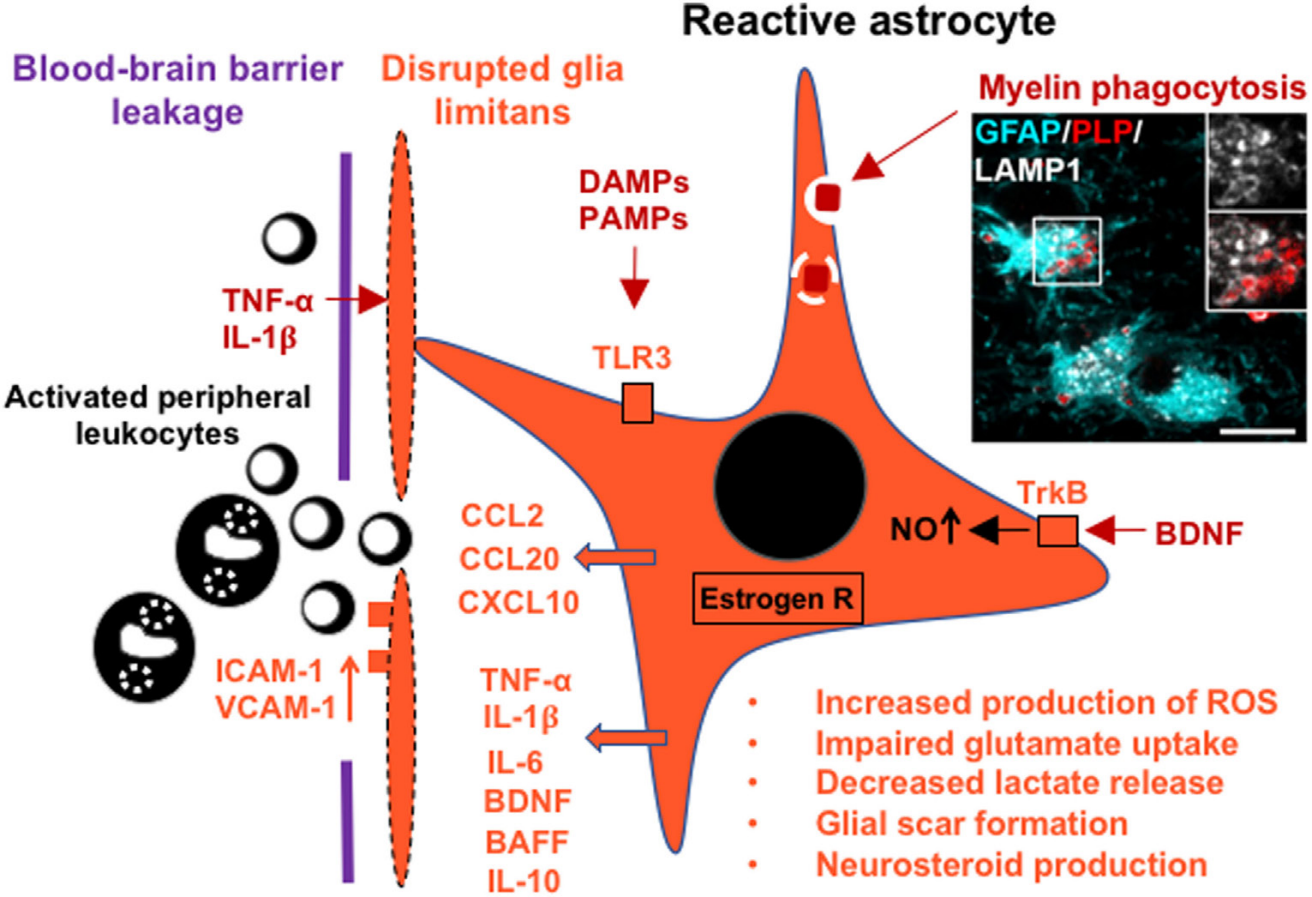
Schematic of the glia limitans and a reactive astrocyte in multiple sclerosis (MS), indicating secretion of cytokines, recruitment of leukocytes across the blood–brain barrier, and upregulation of selected receptors that contribute to astrocyte responses and metabolic changes. Inset image shows a reactive hypertrophic astrocyte at the active rim of an MS lesion containing myelin debris within lysosomal ring structures [glial fibrillary acidic protein (GFAP), cyan; myelin proteolipid protein (PLP), red; lysosomal-associated membrane protein 1 (LAMP1), white]. Scale bar = 10 µm. The inset image was reproduced from Figure 1 of Ponath et al. (17) with the permission of Brain.

Astrocytes also constitutively express low amounts of MHC-II and the adhesion molecules LFA-1 (CD11a) and ICAM-1 (CD54) (59). Stimulation with IFN-γ alone or in combination with TNF-α upregulates MHC-II, adhesion molecules, and co-stimulatory molecules B7-1 (CD80) and B7-2 (CD86) (60). Functional studies have shown that IFN-γ-treated murine astrocytes act as weak antigen-presenting cells, moderately activating CD4^+^ and CD8^+^ T cells. In contrast, cytokine-treated human astrocytes were not able to induce proliferation of encephalitogenic T cells, presumably because of lack of additional proliferation-inducing factors (59), suggesting interspecies differences in astrocytes. Furthermore, in the inflamed CNS, reactive astrocytes may contribute to B cell survival, maturation, and proliferation through production of B cell-activating factor of the TNF family (BAFF) (61–63). Other soluble factors secreted by astrocytes, such as IL-6 and IL-15, also support B cell survival (61). Finally, stimulation of astrocytes with cytokines diminishes their homeostatic and metabolic functions, resulting in impaired glutamate uptake, which may cause excitotoxicity, and in metabolic uncoupling from axons/neurons due to decreased release of lactate (23, 24, 64–66) (Figure 1).

Reactive astrocytes have recently been categorized according to their transcriptome profiles as “A1” or “A2,” in analogy to the “M1” and “M2” phenotype categories for macrophages (67). A1-type astrocytes, for which complement component 3 is an identifying marker, are induced by inflammation (67, 68), are abundant in MS and neurodegenerative diseases, including Alzheimer’s and Parkinson’s disease, and secrete a neurotoxin that has not yet been identified (67). In contrast, A2-type astrocytes are induced by ischemia and express neurotrophic factors (67). The concept of M1/M2 polarization is now considered an oversimplification and has been abandoned in favor of multiple, complex polarization states that do not necessarily align with a one-dimensional M1–M2 spectrum (69). Thus, while defining these phenotypes is an important step, reactive astrocytes may also exceed the A1–A2 dichotomy and assume a range of profiles with mixed A1 and A2 features (70). It has been proposed that although reactive astrocytes share common properties, they also display unique cellular and molecular features that are specific to different neuropathologies (70, 71). Moreover, distinct astrocytic phenotypes may coexist or develop sequentially during different phases of a pathological process: reactive astrocytes may first produce pro-inflammatory cytokines and reactive oxygen species in conjunction with hypertrophy and proliferation. In a second phase, astrocytes may promote anti-inflammatory and neuroregenerative functions through astrocyte-derived neurotrophic factors (72).

Thus, reactive astrocytes can mount powerful inflammatory responses that drive leukocyte recruitment to the CNS and thereby contribute to a successful defense against pathogens. Moreover, reactive astrocytes may change their response profiles over time, resulting in the secretion of anti-inflammatory cytokines and neurotrophins (72, 73). Astrocytosis can also aid in BBB repair and, depending on the severity of the injury, lead to the formation of glial scars that isolate the inflamed area, restrict damage and provide structural support (25).

## The Reactive Astrocyte in MS Lesion Pathology

Astrocytes are increasingly recognized as cells that critically contribute to the development of MS lesions. Previously, astrocytes were believed to react only at a late, post-inflammatory stage by forming a glial scar, but are now considered early and active players in lesion pathology (16, 17). In active lesions, astrocytes assume a hypertrophic morphology, characterized by massive enlargement of the cell soma and reduced process density (16). Typically, pronounced astroglial hypertrophy is indicative of substantial tissue injury and might be caused in MS lesions by oligodendrocyte loss and the resulting disruption of astrocyte–oligodendrocyte networks (16, 28). In addition, hypertrophic astrocytes may themselves sustain substantial damage that leads to retraction or loss of glia limitans from the basal lamina around blood vessels, presumably further increasing access of immune cells to the CNS (16) (Figure 1).

Reactive astrocytes are present in the active margins of demyelinating lesions and extend into adjacent, normal-appearing white matter (NAWM), suggesting that they are early contributors to lesion development (16, 17). This view is supported by the observation in murine experimental autoimmune encephalomyelitis (EAE), an inflammatory demyelinating model of MS, that astrocytes in nascent lesions become activated before significant immune cell infiltration into the parenchyma takes place (74–76). Furthermore, we have shown that hypertrophic astrocytes at the leading edge of actively demyelinating MS lesions contain myelin debris (17). We have demonstrated that this myelin uptake induces astroglial NF-κB signaling and secretion of cell-recruiting chemokines. Therefore, we hypothesized that uptake of damaged myelin by astrocytes may be an early trigger for their activation, leading to astrocyte-mediated influx of leukocytes at the very beginning of lesion development (17) (Figure 1). Given that astrocytes in MS lesions express MHC class II and co-stimulatory molecules CD80 and CD86 (77, 78), it is tempting to speculate that myelin phagocytosis by astrocytes results in the presentation of myelin antigens to T cells. However, since stimulated human astrocytes in culture fail to induce, and even inhibit, proliferation of T cells (79), we consider it unlikely that astrocytes act as competent antigen-presenting cells in MS lesions.

In MS, hypertrophic astrocytes express chemokines and cell adhesion molecules associated with macrophage/microglia and lymphocyte recruitment into the parenchyma (80–82). Their functional relevance to leukocyte recruitment has been well documented in EAE. For example, in mice with a conditional, astrocyte-specific gene deletion of CCL2, induction of EAE resulted in a less severe disease course with fewer macrophage and T cell infiltrates, and less activation of astrocytes and microglia (83). Similarly, mice with a genetic deletion of all ICAM-1 isoforms showed marked attenuation of EAE, with minimal cellular infiltration and demyelination in the spinal cord (84). Conversely, astrocyte-mediated recruitment of microglia to demyelinating lesions is also of benefit, as demonstrated in a demyelination model using the oligodendrocyte toxin cuprizone, which does not disrupt the BBB or involve peripheral immune cell infiltration (85). Genetic ablation of astrocytes in mice treated with cuprizone prevented the recruitment of microglia cells to the site of demyelination, leading to delayed removal of myelin debris, impaired remyelination, and reduced proliferation of oligodendrocyte precursor cells (86). Thus, activated astrocytes are key regulators for the removal of damaged myelin, which is needed before remyelination can take place (86).

In addition, BAFF production by reactive astrocytes may contribute to the pathogenesis of MS by promoting B cell survival and proliferation in the CNS (61, 62). BAFF levels were shown to be increased in the CSF of MS patients compared to healthy controls (87). Moreover, BAFF mRNA was strongly upregulated in MS lesions, comparable to levels observed in lymphatic tissues, and BAFF was found to be expressed in reactive astrocytes, adjacent to inflammatory cells that expressed BAFF receptors (63). Given the continuous presence of antigen-experienced B cell clones in the CNS of MS patients (88) and the development of meningeal B cell follicles in progressive MS (89), astroglial production of BAFF may be a major factor to sustain these cells and to drive B cell-related pathology.

Reactive astrocytes likely contribute to tissue damage in MS through impaired glutamate handling and redox homeostasis. Glutamate concentrations were shown to be elevated in acute lesions of MS patients using *in vivo* MR spectroscopy (90). Moreover, a GWAS has linked specific risk variants associated with glutamate metabolism to increased cortical glutamate concentrations and poor disease outcomes in MS patients (91). In EAE, disease severity as well as oligodendrocyte and neuronal death were ameliorated through treatment with antagonists to the AMPA/Kainate or NMDA type of glutamate receptors (23, 24).

A recent study in a chronic progressive model of EAE has shown that astrocytes produce and are stimulated by the sphingolipid lactosylceramide (LacCer) (7). LacCer induces production of pro-inflammatory cytokines and iNOS in astrocytes and promotes pathology during experimental spinal cord injury (92). In EAE, LacCer was found to control the recruitment and activation of microglia and CNS-infiltrating monocytes by astrocytes. In addition, inhibition of LacCer synthesis suppressed CNS innate immunity and neurodegeneration. Finally, LacCer and the LacCer synthase β-1,4-galactosyltransferase 6 (B4GALT6) were detected in reactive astrocytes within MS lesions (7), suggesting that the B4GALT6-LacCer pathway is relevant to human disease.

Although reactive astrocytes drive inflammatory and neurotoxic responses in MS lesions, they may also dampen inflammation and promote neuroprotection and lesion repair. A factor produced by astrocytes and neurons in the normal CNS, which has CNS-trophic effects, is BDNF (93, 94). In EAE, astrocyte-specific deletion of BDNF resulted in a more severe clinical course with increased axonal loss (95). Moreover, in the cuprizone mouse model, enhanced BDNF production by astrocytes, induced by stimulation of metabotropic glutamate receptors, resulted in enhanced remyelination (96). However, a separate study demonstrated that signaling through the BDNF receptor TrkB in astrocytes leads to production of nitric oxide (NO) (97). EAE induced in mice with astrocyte-specific genetic deletion of TrkB had ameliorated disease severity, concomitant with reduced expression of astrocytic and lesional iNOS (97). These data indicate that BDNF released by astrocytes not only elicits neuroprotective effects in other cell types but also stimulates production and release of toxic NO in astrocytes themselves. In MS lesions, BDNF is primarily present in immune cells and reactive astrocytes (98), while the BDNF receptor TrkB was strongly upregulated in reactive astrocytes and in neurons in the immediate lesion vicinity (98). This suggests a possible dual protective and degenerative role for BDNF.

Astrocytes are susceptible to neurosteroids, such as estrogen and DHEA, which downregulate pro-inflammatory responses in reactive astrocytes (99–101). This mechanism plays a significant role in EAE where treatment of mice with an estrogen receptor-α (ERα) ligand substantially ameliorated clinical symptoms, inflammatory infiltrates, and axonal loss (102, 103). These beneficial effects were mediated entirely through ERα expressed by astrocytes, as they were abolished in EAE induced in mice with conditional, astrocyte-specific deletion of ERα (103). In MS lesions, ERα, aromatase, an enzyme involved in estrogen synthesis, and progesterone receptor were found to be upregulated in reactive astrocytes (104), suggesting that neurosteroid synthesis by reactive astrocytes as well as astrocytic responses to neurosteroids are part of an endogenous protective mechanism. On the other hand, a recent study found that the neurosteroids allopregnanolone and DHEA were substantially downregulated in EAE and in NAWM of autopsied MS tissue (105). Provided that astrocytes are the main steroidogenic cells in the brain (38), these data may point toward impaired synthesis of both neurosteroids by astrocytes in MS.

In addition, TLR signaling may play a neuroprotective role in EAE and by extension, in MS, although this effect might not be astrocyte-specific. Systemic administration of the TLR3 agonist polyinosinic:polycytidylic acid (poly I:C) in EAE suppresses relapsing demyelination through induction of IFN-β and other immune regulatory effects (106). Furthermore, TLR4 knockout mice exhibited more severe EAE symptoms than wild-type mice, associated with increased priming of encephalitogenic Th17 cells (107). In MS lesions, TLR3 and 4 are expressed by microglia and astrocytes, where astroglial TLR expression is particularly prominent at later stages of inflammation, which may be instrumental in mitigating inflammation and promote tissue repair (56, 58).

Furthermore, following acute inflammation and demyelination, hypertrophic astrocytes eventually form a glial scar in the center of white matter lesions (25). While scars have been considered as barriers to tissue regeneration (16), they also provide beneficial features and contribute to recovery from CNS insults (25). For example, glial scars support demyelinated axons, help restore BBB function, and confine inflamed areas, preventing the spread of immune cells and toxic levels of extracellular ions, metabolites, or DAMPs into healthy tissues or areas of repair (16, 25).

Recent studies have implicated gut microbiota in immunological disorders including MS and its animal model, EAE (108, 109). The microbiome has emerged as a regulator of BBB integrity, where the absence of normal gut flora leads to disorganization of tight junctions in endothelial cells (110), and the production of short-chain fatty acids by bacteria corrects BBB dysfunction (111, 112). However, to date, astrocytes have not been found to mediate these effects.

## Signaling Pathways in Astrocytes

**Figure 2:**
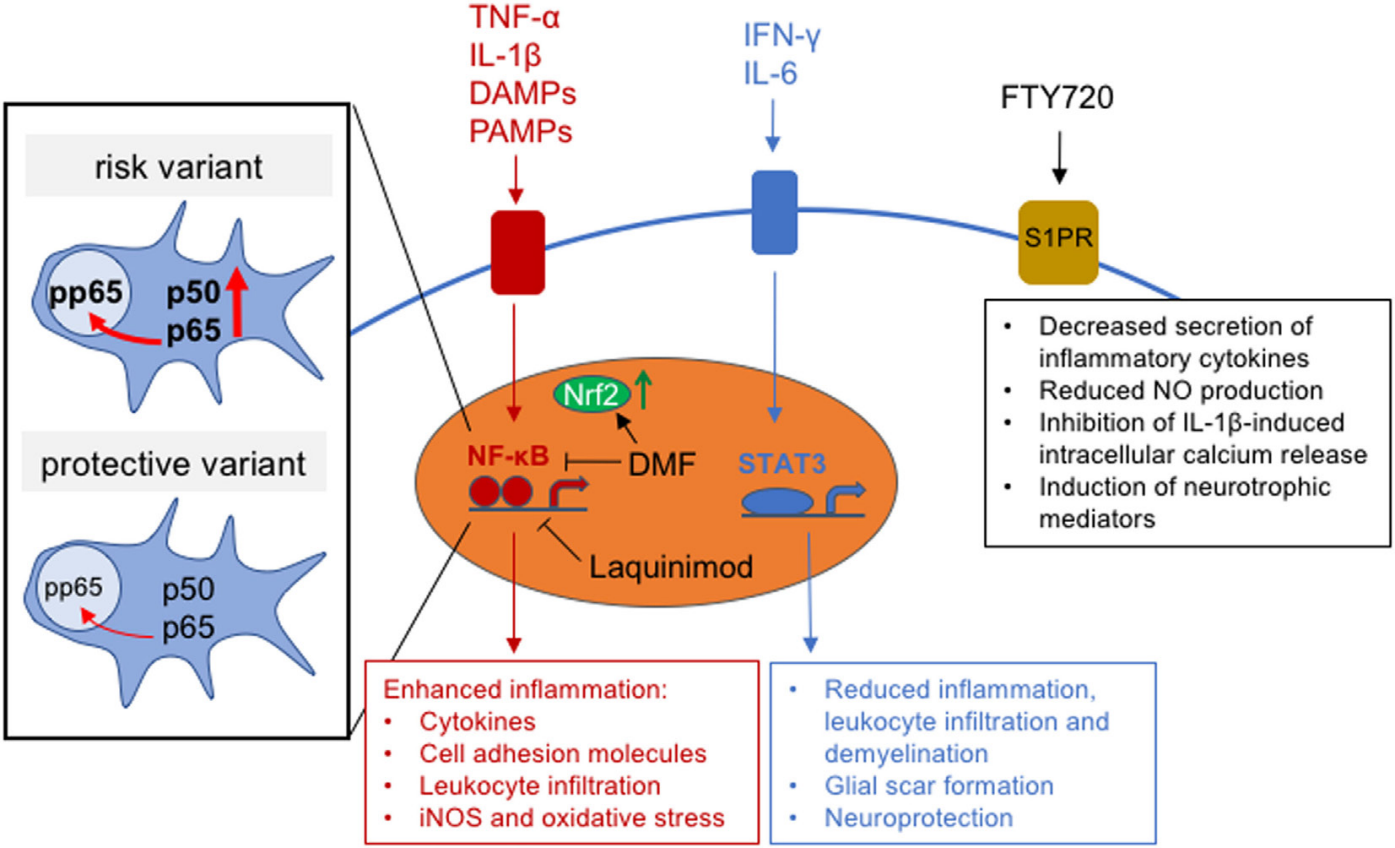
Intracellular astrocytic signaling pathways and effects of multiple sclerosis (MS) treatments on signaling function. Inset shows schematic of the effect of MS risk variant, rs7665090, on NF-κB subunit expression and NF-κB signaling.

Astrocyte reactivity is regulated by key canonical signaling cascades, among which the NF-κB pathway is pivotal for establishing neuroinflammation (113) (Figure 2). NF-κB is a master regulator of innate and adaptive immunity that controls cell survival, differentiation, and proliferation (114). Astrocytic NF-κB signaling is directly activated through stimulation with the pro-inflammatory cytokines TNF-α and IL-1β (113), through TLR signaling and various other agents including phagocytosed myelin, mitogens, and free radicals (17, 113, 115, 116). NF-κB signaling in astrocytes plays a critical role for initiating and maintaining inflammation in the CNS. Transgenic mice with astrocyte-specific inactivation of NF-κB display dramatic amelioration of tissue damage and clinical impairment following induction of EAE, spinal cord injury, or ischemic retinal injury compared to wild-type mice (117–119). Similarly, ablation of IL-17-induced Act1 signaling in astrocytes, which abolishes IL-17-mediated NF-κB activation, reduces the recruitment of lymphocytes and macrophages and markedly ameliorates disease severity in EAE (120).

Interestingly, microbial flora and its products have been shown to control NF-κB signaling through conversion of dietary tryptophan into agonists of aryl hydrocarbon receptor (AHR), which is highly expressed by astrocytes (121). AHR suppresses the classical activation pathways of NF-κB through competitive binding to the NF-κB subunit p65 (122). Induction of EAE in mice with astrocyte-specific genetic knockout of AHR [glial fibrillary acidic protein (GFAP)–AHR^−^] resulted in increased expression of chemokines, cytokines and pro-inflammatory markers and an exacerbated disease course compared to wild type animals. Moreover, mice fed with a tryptophan-depleted diet exhibited a more severe EAE course, which could not be reversed by addition of tryptophan in GFAP–AHR^−^ mice. In MS, *AHR* expression was upregulated in active and chronic MS lesions and localized to GFAP^+^ astrocytes; however, this might not translate into AHR-dependent downregulation of astrocyte activation, because expression of the AHR transcriptional target *CYP1B1* was decreased in MS lesions and NAWM, suggesting that this pathway is defective in MS (121).

We have recently shown that an MS risk variant, rs7665090, which increases NF-κB signaling in lymphocytes (123), substantially affects astrocyte reactivity in cell culture and MS white matter lesions (81). Astrocytes derived from induced pluripotent stem cells, obtained from MS patients carrying the risk variant, showed increased NF-κB activation, chemokine and cell adhesion molecule expression, as well as impaired glutamate uptake and reduced lactate release. In addition, the risk variant was associated with significantly higher numbers of infiltrating lymphocytes in white matter MS lesions and with an increased lesion load on MRI in MS patients (81). Therefore, this NF-κB-relevant risk variant promotes pro-inflammatory changes in astrocytes that might help target aberrant immune responses to the CNS. This challenges the view that MS is mediated solely through dysregulation of lymphocytes and highlights the importance of astroglial NF-κB signaling for lesion formation (81) (Figure 2).

An important but less elucidated signaling cascade for regulation of astrocyte activation in MS is the STAT3 pathway (Figure 2). STAT3 activity is generally upregulated in response to CNS inflammation and damage (124). In astrocytes, STAT3 signaling is induced by both pro- and anti-inflammatory molecules, including IFN-γ and cytokines of the IL-6 family, that bind to the gp130 cell-surface receptor to induce STAT3 phosphorylation (124–127). STAT3 signaling in astrocytes plays a beneficial role in CNS inflammation, as demonstrated in mice with conditional, astrocyte-specific knockout of STAT3, where spinal cord injury lesions exhibited increased demyelination, contained more infiltrating dendritic cells, and had attenuated astrocyte hypertrophy and glial scar formation (128, 129). Similarly, in EAE, disease severity was exacerbated in mice with astrocyte-specific knockout of the STAT3-activating gp130 signal transducer, with larger areas of demyelination and increased infiltration of reactive T-lymphocytes (130). Moreover, activated astrocytes have been shown to provide neuronal protection *via* ERK (131) and/or STAT3 signaling during inflammation (132). This was demonstrated in an *in vivo* model of acute LPS/IFN-γ-induced neuroinflammation, where STAT3 and ERK signaling induced IL-6 production, which protected against neuronal apoptosis (133). Despite its importance as a neurotrophin in the CNS, IL-6 is also known to promote MS lesion development when produced in excess (134, 135). Specifically, IL-6 inhibits differentiation of naïve T cells into regulatory T cells and promotes their differentiation into Th17 helper cells, which are considered to be major mediators of MS pathology (134). Thus, IL-6 levels above or below a certain threshold may determine its role as either a growth factor and activator of STAT3-mediated anti-inflammatory function, or as a suppressor of regulatory T cell differentiation and enhancer of pro-inflammatory helper T cell activity. Therefore, understanding the dynamics of IL-6 production in CNS lesions may help to predict the effectiveness of STAT3 signaling as a suppressor of lesion pathology.

## Direct Modulation of Reactive Astrocyte Activity by MS Treatments

As discussed above, activated astrocytes play multiple pivotal roles during inflammation, including regulation of leukocyte trafficking, release of neurotoxic factors, confinement of inflammation, and promotion of neuroprotection and tissue repair. This makes astrocytes obvious therapeutic targets in MS. Ideally, such treatments would take into account the multi-functionality of astrocytes to block detrimental responses and/or enhance regenerative properties. Current MS therapies that are known to cross the BBB and modulate astrocyte function are laquinimod, which is currently being developed as an MS treatment, dimethyl fumarate (DMF; Tecfidera^®^) and fingolimod (FTY720; Gilenya^®^)/siponimod. In clinical studies, siponimod and laquinimod have shown a positive impact on progressive MS and brain atrophy, respectively. Since progressive MS is believed to be driven in part by chronic glial activation, these studies provide circumstantial evidence that astrocyte activation may contribute to progressive pathology. Below, we provide details of how each of these compounds impacts astrocytes.

### Laquinimod

Laquinimod is a small quinolone derivative of the immunomodulatory compound linomide. Laquinimod was initially tested in RRMS, where it led to moderate effects on the reduction of relapse rates as a primary study endpoint. However, significant effects were observed on brain atrophy and disease progression (136). This led to a clinical trial of laquinimod in primary progressive MS (ARPEGGIO trial), which is still ongoing (137).

While the precise molecular targets of laquinimod are not well defined, recent data suggests that laquinimod activates genes associated with the transcription factor aryl hydrocarbon receptor (AHR, see above) (138). AHR target genes encode for drug-metabolizing enzymes and proteins controlling cell proliferation, differentiation, and apoptosis (139, 140). Additionally, cross talk between AHR and other signaling pathways, including epidermal growth factor receptor (EGFR) (141, 142), protein kinase A (PKA) (143, 144), and NF-κB signaling (144, 145), has been reported (146). Accordingly, AHR modulates the differentiation and function of many cell populations, several of which play an important role in neuroinflammation. In mouse EAE, laquinimod exerts effects on the peripheral immune system, where it downregulates pro-inflammatory T cell responses (147, 148), and on CNS cells. Genetic deletion of AHR in the immune system fully abrogated the treatment effect of laquinimod on EAE, while deletion of AHR in the CNS partially abrogated this effect (138). In a separate study, laquinimod markedly reduced NF-κB signaling and pro-inflammatory activation of astrocytes, but not of microglia *in vitro* (Figure 2). In the cuprizone model of demyelination, laquinimod prevented demyelination, microglial activation, T cell infiltration, and axonal transection; this effect was attributed to *in vivo* attenuation of NF-κB signaling in astrocytes (149). Laquinimod exhibits additional modes of action including neuroprotection, as demonstrated in EAE, where conditional deletion of BDNF in myeloid and T cells partially abrogated the beneficial effect of laquinimod. Similarly, laquinimod treatment of MS patients was found to increase expression of BDNF in serum (150). Taken together, current data suggests that laquinimod exerts effects on multiple cell types during CNS inflammation. A key mechanism mediated by laquinimod is the downregulation of pro-inflammatory responses in astrocytes. Thus, the beneficial effects of laquinimod on brain atrophy and disability progression in MS patients may at least partially be explained by its direct effect on astrocytes.

### Dimethyl Fumarate

Dimethyl fumarate is the methyl ester of fumaric acid and was FDA-approved for the treatment of relapsing forms of MS in 2013. In placebo-controlled clinical trials, Tecfidera^®^ reduced the relapse rate in MS patients by approximately 50% and disability progression by 38% in one trial but not in a separate, parallel trial (151, 152). The effect of Tecfidera^®^ on SPMS is unclear, as a phase III clinical trial was initiated but terminated early due to restructuring of the drug’s manufacturer, Biogen (153). DMF activates the Nrf2 transcription factor, which targets antioxidant response element (ARE) genes coding for antioxidant enzymes that reduce oxidative stress (154). DMF induces Nrf2 through glutathione depletion and direct binding to the Nrf2 repressor Kelch-like ECH-associated protein 1 (KEAP1) (155–158). Moreover, DMF acts as a potent inhibitor of NF-κB signaling (159) and has been shown to modify DMF-sensitive cysteine residues in human T cells, which inhibits T cell activation (160) (Figure 2).

In the peripheral immune system, DMF reduces lymphocyte counts, in particular cytotoxic and effector T cells, and inhibits activation of antigen-presenting cells (161). In the CNS, a major effect of DMF is the upregulation of Nrf2 in astrocytes, which is protective against oxidative injury *via* upregulation of oxidative stress-induced growth inhibitor 1 (162). This effect might ameliorate astrocytic damage in active lesions, including the retraction of perivascular astrocyte end feet along basal lamina (16), to reduce leakage across the BBB and the cortical surface (163). DMF also inhibits secretion of pro-inflammatory cytokines and chemokines by astrocytes and microglial cells, independent of changes in antioxidant gene expression (164). Therefore, in addition to its effect on the peripheral immune system, DMF has a direct impact on the CNS that involves protective and anti-inflammatory effects on astrocytes.

### Fingolimod and Siponimod

FTY720/fingolimod (2-amino-2[2-(4-octylphenyl)ethyl] propane-1,3-diol hydrochloride; Gilenya^®^) is a non-specific sphingosine-I phosphate (S1P) modulator. In clinical trials with RRMS patients, FTY720 reduced the annualized relapse rate by 48–55% and decreased risk of disability progression by 28% in one study, while having no significant effect on disability in another (165, 166). With regards to primary progressive MS, a recent trial demonstrated that FTY720 had no beneficial effects on disability progression or whole-brain atrophy (167). However, Siponimod, a selective modulator of the S1P_1_ and S1P_5_ receptors, did slow disability progression in SPMS in a phase III clinical trial (168).

The main effect of FTY720 on the peripheral immune system is the internalization and degradation of the S1P receptor on lymphocytes, which results in impaired responses to the S1P gradient in lymph nodes and prevents lymphocyte egress (169, 170). In the CNS, S1P receptors play a number of roles in brain cell function, including astrocyte proliferation and migration (171, 172), oligodendrocyte differentiation and survival (173, 174), and neurite outgrowth and neurogenesis (175–177). The mechanism most relevant to MS and its animal model, EAE, involves S1P_1_ receptor signaling in astrocytes, which has been demonstrated in conditional null mouse mutants lacking S1P_1_ in astrocytes. When induced to develop EAE, these mice showed a substantial reduction in disease severity, which was not further affected through additional FTY720 treatment, suggesting that the main effect of FTY720 in EAE involves modulation of astrocyte function but not the arrest of lymphocytes in lymph nodes (178). In contrast, conditional deletion of S1P_1_ in neuronal cell lineages had no impact on EAE severity or the efficacy of FTY720 to suppress EAE. Astrocytes mainly express S1P_1_ and S1P_3_ as well as other subtypes at low levels (171, 179). Expression of both receptors is markedly increased in reactive astrocytes in active and chronic MS lesions. Moreover, treatment of cultured human astrocytes with FTY720 limits secretion of inflammatory cytokines (180), reduces NO production (181), induces neurotrophic mediators, and inhibits TNF-α-induced inflammatory gene expression (182). Modulation of astrocytic intracellular pathway function induced by FTY720 includes enhanced expression of calcium-regulating proteins and inhibition of calcium release induced by the pro-inflammatory mediator IL-1β (183) (Figure 2). This data implicates S1P_1_ signaling in astrocytes as a major contributor to the pathogenesis of EAE and as the main therapeutic target of FTY720 (184). Thus, the efficacy of Siponimod, a modulator of the S1P_1_ and S1P_5_ receptors, in slowing disability accumulation in SPMS may be mediated through its direct effect on astrocytes.

Other MS therapies, such as teriflunomide (Aubagio^®^) (185, 186) and IFN-β (Avonex^®^, Betaseron^®^, Rebif^®^), have been shown to inhibit astroglial immune responses, the latter by inducing astroglial expression of AHR (121). However, teriflunomide and IFN-β have limited or no BBB penetrance (187, 188), making it unlikely that these drugs exert continuous, direct effects on CNS-resident cells.

## Conclusion

Astrocytes play an instrumental role in the formation of MS lesions through a multitude of functional changes associated with their activation. Astrocytes are early responders in nascent white matter lesions, are the main recruiters of lymphocytes, and act themselves as immunocompetent cells that contribute to innate immunity. Moreover, astrocytes not only can adopt a neurotoxic phenotype, but also confine inflammation through scar formation and can promote neuroprotection and tissue repair. Astrocytic dysfunction associated with a genetic MS risk variant further suggests that astrocyte-mediated processes are causative in lesion pathology. Thus, while MS is driven by dysfunction of the peripheral immune system, CNS cells such as astrocytes may contribute to MS pathology by targeting dysregulated immune responses to the CNS. Finally, MS medications that impact astrocytes have shown efficacy in both relapsing–remitting and phase III clinical trials of progressive MS, providing further circumstantial evidence that activation of astrocytes contributes to both pathologies.

## Author Contributions

GP, CP and DP wrote the manuscript.

## Conflict of Interest Statement

The authors declare that the research was conducted in the absence of any commercial or financial relationships that could be construed as a potential conflict of interest.

## References

[1] Nylander A Hafler DA. Multiple sclerosis. J Clin Invest (2012) 122:1180–8. 10.1172/JCI58649 22466660 PMC3314452

[2] Wallin MT The prevalence of multiple sclerosis in the united states: a population-based healthcare database approach. ECTRIMS Online Libr (2017) 26:199999.

[3] Frohman EM Racke MK Raine CS Multiple sclerosis – the plaque and its pathogenesis. N Engl J Med (2006) 354:942–55. 10.1056/NEJMra052130 16510748

[4] Hafler DA. Multiple sclerosis. J Clin Invest (2004) 113:788–94. 10.1172/JCI21357 15067307 PMC362131

[5] Lassmann H van Horssen J Mahad D Progressive multiple sclerosis: pathology and pathogenesis. Nat Rev Neurol (2012) 8:647–56. 10.1038/nrneurol.2012.168 23007702

[6] Kutzelnigg A Lucchinetti CF Stadelmann C Bruck W Rauschka H Bergmann M Cortical demyelination and diffuse white matter injury in multiple sclerosis. Brain (2005) 128:2705–12. 10.1093/brain/awh641 16230320

[7] Mayo L Trauger SA Blain M Nadeau M Patel B Alvarez JI Regulation of astrocyte activation by glycolipids drives chronic CNS inflammation. Nat Med (2014) 20:1147–56. 10.1038/nm.3681 25216636 PMC4255949

[8] Soelberg Sorensen P. Safety concerns and risk management of multiple sclerosis therapies. Acta Neurol Scand (2017) 136:168–86. 10.1111/ane.12712 27891572

[9] Sawcer S Hellenthal G Pirinen M Spencer CC Patsopoulos NA Moutsianas L Genetic risk and a primary role for cell-mediated immune mechanisms in multiple sclerosis. Nature (2011) 476:214–9. 10.1038/nature10251 21833088 PMC3182531

[10] Beecham AH Patsopoulos NA Xifara DK Davis MF Kemppinen A Cotsapas C Analysis of immune-related loci identifies 48 new susceptibility variants for multiple sclerosis. Nat Genet (2013) 45:1353–60. 10.1038/ng.2770 24076602 PMC3832895

[11] Farh KK-H Marson A Zhu J Kleinewietfeld M Housley WJ Beik S Genetic and epigenetic fine mapping of causal autoimmune disease variants. Nature (2015) 518:337–43. 10.1038/nature13835 25363779 PMC4336207

[12] Ascherio A. Environmental factors in multiple sclerosis. Expert Rev Neurother (2013) 13:3–9. 10.1586/14737175.2013.865866 24289836

[13] Hernandez AL Kitz A Wu C Lowther DE Rodriguez DM Vudattu N Sodium chloride inhibits the suppressive function of FOXP3+ regulatory T cells. J Clin Invest (2015) 125:4212–22. 10.1172/JCI81151 26524592 PMC4639983

[14] O’Gorman C Lucas R Taylor B. Environmental risk factors for multiple sclerosis: a review with a focus on molecular mechanisms. Int J Mol Sci (2012) 13:11718–52. 10.3390/ijms130911718 23109880 PMC3472772

[15] Kuhlmann T Ludwin S Prat A Antel J Bruck W Lassmann H. An updated histological classification system for multiple sclerosis lesions. Acta Neuropathol (2017) 133:13–24. 10.1007/s00401-016-1653-y 27988845

[16] Brosnan CF Raine CS. The astrocyte in multiple sclerosis revisited. Glia (2013) 61:453–65. 10.1002/glia.22443 23322421

[17] Ponath G Ramanan S Mubarak M Housley W Lee S Sahinkaya FR Myelin phagocytosis by astrocytes after myelin damage promotes lesion pathology. Brain (2017) 140:399–413. 10.1093/brain/aww298 28007993 PMC5841057

[18] Sofroniew MV. Astrocyte barriers to neurotoxic inflammation. Nat Rev Neurosci (2015) 16:249–63. 10.1038/nrn3898 25891508 PMC5253239

[19] Farina C Aloisi F Meinl E. Astrocytes are active players in cerebral innate immunity. Trends Immunol (2007) 28:138–45. 10.1016/j.it.2007.01.005 17276138

[20] Ransohoff RM Brown MA. Innate immunity in the central nervous system. J Clin Invest (2012) 122:1164–71. 10.1172/JCI58644 22466658 PMC3314450

[21] Karadottir R Cavelier P Bergersen LH Attwell D. NMDA receptors are expressed in oligodendrocytes and activated in ischaemia. Nature (2005) 438:1162–6. 10.1038/nature04302 16372011 PMC1416283

[22] Micu I Jiang Q Coderre E Ridsdale A Zhang L Woulfe J NMDA receptors mediate calcium accumulation in myelin during chemical ischaemia. Nature (2006) 439:988–92. 10.1038/nature04474 16372019

[23] Pitt D Werner P Raine CS. Glutamate excitotoxicity in a model of multiple sclerosis. Nat Med (2000) 6:67–70. 10.1038/71555 10613826

[24] Smith T Groom A Zhu B Turski L. Autoimmune encephalomyelitis ameliorated by AMPA antagonists. Nat Med (2000) 6:62–6. 10.1038/71548 10613825

[25] Sofroniew MV Vinters HV Astrocytes: biology and pathology. Acta Neuropathol (2010) 119:7–35. 10.1007/s00401-009-0619-8 20012068 PMC2799634

[26] von Bartheld CS Bahney J Herculano-Houzel S. The search for true numbers of neurons and glial cells in the human brain: a review of 150 years of cell counting. J Comp Neurol (2016) 524:3865–95. 10.1002/cne.24040 27187682 PMC5063692

[27] Horng S Therattil A Moyon S Gordon A Kim K Argaw AT Astrocytic tight junctions control inflammatory CNS lesion pathogenesis. J Clin Invest (2017) 127:3136–51. 10.1172/JCI91301 28737509 PMC5531407

[28] Orthmann-Murphy JL Abrams CK Scherer SS. Gap junctions couple astrocytes and oligodendrocytes. J Mol Neurosci (2008) 35:101–16. 10.1007/s12031-007-9027-5 18236012 PMC2650399

[29] Ventura R Harris KM. Three-dimensional relationships between hippocampal synapses and astrocytes. J Neurosci (1999) 19:6897. 10436047 10.1523/JNEUROSCI.19-16-06897.1999 PMC6782870

[30] Bezzi P Volterra A. A neuron-glia signalling network in the active brain. Curr Opin Neurobiol (2001) 11:387–94. 10.1016/S0959-4388(00)00223-3 11399439

[31] Santello M Volterra A Neuroscience: astrocytes as aide-memoires. Nature (2010) 463:169–70. 10.1038/463169a 20075911

[32] Chung W-S Allen NJ Eroglu C. astrocytes control synapse formation, function, and elimination. Cold Spring Harb Perspect Biol (2015) 7:a020370. 10.1101/cshperspect.a020370 25663667 PMC4527946

[33] Lafourcade C Ramírez JP Luarte A Fernández A Wyneken U. MiRNAs in astrocyte-derived exosomes as possible mediators of neuronal plasticity. J Exp Neurosci (2016) 10:1–9. 10.4137/JEN.S39916 27547038 PMC4978198

[34] Magistretti PJ. Neuron-glia metabolic coupling and plasticity. J Exp Biol (2006) 209:2304–11. 10.1242/jeb.02208 16731806

[35] Masutani H Bai J Kim YC Yodoi J. Thioredoxin as a neurotrophic cofactor and an important regulator of neuroprotection. Mol Neurobiol (2004) 29:229–42. 10.1385/MN:29:3:229 15181236

[36] Anderson CM Bergher JP Swanson RA. ATP-induced ATP release from astrocytes. J Neurochem (2004) 88:246–56. 10.1111/j.1471-4159.2004.02204.x 14675168

[37] Akwa Y Sananès N Gouézou M Robel P Baulieu EE Le Goascogne C. Astrocytes and neurosteroids: metabolism of pregnenolone and dehydroepiandrosterone. Regulation by cell density. J Cell Biol (1993) 121:135–43. 10.1083/jcb.121.1.135 8458866 PMC2119772

[38] Zwain IH Yen SS. Neurosteroidogenesis in astrocytes, oligodendrocytes, and neurons of cerebral cortex of rat brain. Endocrinology (1999) 140:3843–52. 10.1210/endo.140.8.6907 10433246

[39] Reddy DS Neurosteroids: endogenous role in the human brain and therapeutic potentials. Prog Brain Res (2010) 186:113–37. 10.1016/B978-0-444-53630-3.00008-7 21094889 PMC3139029

[40] Schumacher M Hussain R Gago N Oudinet J-P Mattern C Ghoumari AM. Progesterone synthesis in the nervous system: implications for myelination and myelin repair. Front Neurosci (2012) 6:10. 10.3389/fnins.2012.00010 22347156 PMC3274763

[41] Kipper-Galperin M Galilly R Danenberg HD Brenner T Dehydroepiandrosterone selectively inhibits production of tumor necrosis factor α and Interlukin-6 in astrocytes. Int J Dev Neurosci (1999) 17:765–75. 10.1016/S0736-5748(99)00067-2 10593612

[42] John GR Lee SC Brosnan CF. Cytokines: powerful regulators of glial cell activation. Neuroscientist (2003) 9:10–22. 10.1177/1073858402239587 12580336

[43] Cannella B Raine CS. The adhesion molecule and cytokine profile of multiple sclerosis lesions. Ann Neurol (1995) 37:424–35. 10.1002/ana.410370404 7536402

[44] Bechmann I Mor G Nilsen J Eliza M Nitsch R Naftolin F. FasL (CD95L, Apo1L) is expressed in the normal rat and human brain: evidence for the existence of an immunological brain barrier. Glia (1999) 27:62–74. 10.1002/(SICI)1098-1136(199907)27:1<62::AID-GLIA7>3.0.CO;2-S 10401633

[45] Choi C Benveniste EN. Fas ligand/Fas system in the brain: regulator of immune and apoptotic responses. Brain Res Brain Res Rev (2004) 44:65–81. 10.1016/j.brainresrev.2003.08.007 14739003

[46] Gimsa U ØRen A Pandiyan P Teichmann D Bechmann I Nitsch R Astrocytes protect the CNS: antigen-specific T helper cell responses are inhibited by astrocyte-induced upregulation of CTLA-4 (CD152). J Mol Med (Berl) (2004) 82:364–72. 10.1007/s00109-004-0531-6 15007511

[47] Jensen CJ Massie A De Keyser J. Immune players in the CNS: the astrocyte. J Neuroimmune Pharmacol (2013) 8:824–39. 10.1007/s11481-013-9480-6 23821340

[48] Rothhammer V Quintana FJ. Control of autoimmune CNS inflammation by astrocytes. Semin Immunopathol (2015) 37:625–38. 10.1007/s00281-015-0515-3 26223505 PMC4618768

[49] Choi SS Lee HJ Lim I Satoh J Kim SU. Human astrocytes: secretome profiles of cytokines and chemokines. PLoS One (2014) 9:e92325. 10.1371/journal.pone.0092325 24691121 PMC3972155

[50] Lee SJ Benveniste EN. Adhesion molecule expression and regulation on cells of the central nervous system. J Neuroimmunol (1999) 98:77–88. 10.1016/S0165-5728(99)00084-3 10430040

[51] Williams WM Castellani RJ Weinberg A Perry G Smith MA. Do β-defensins and other antimicrobial peptides play a role in neuroimmune function and neurodegeneration? Scientific World Journal (2012) 2012:905785. 10.1100/2012/905785 22606066 PMC3346844

[52] Mahida YR Cunliffe RN. Defensins and mucosal protection. Novartis Found Symp (2004) 263:71–7. discussion 77-84, 211-8, 10.1002/0470090480.ch6 15669635

[53] Chao CC Lokensgard JR Sheng WS Hu S Peterson PK. IL-1-induced iNOS expression in human astrocytes via NF-kappa B. Neuroreport (1997) 8:3163–6. 10.1097/00001756-199709290-00031 9331934

[54] Sheng WS Hu S Feng A Rock RB. Reactive oxygen species from human astrocytes induced functional impairment and oxidative damage. Neurochem Res (2013) 38:2148–59. 10.1007/s11064-013-1123-z 23918204 PMC3798006

[55] Farina C Krumbholz M Giese T Hartmann G Aloisi F Meinl E. Preferential expression and function of toll-like receptor 3 in human astrocytes. J Neuroimmunol (2005) 159:12–9. 10.1016/j.jneuroim.2004.09.009 15652398

[56] Bsibsi M Ravid R Gveric D van Noort JM. Broad expression of toll-like receptors in the human central nervous system. J Neuropathol Exp Neurol (2002) 61:1013–21. 10.1093/jnen/61.11.1013 12430718

[57] Jack CS Arbour N Manusow J Montgrain V Blain M McCrea E TLR signaling tailors innate immune responses in human microglia and astrocytes. J Immunol (2005) 175:4320–30. 10.4049/jimmunol.175.7.4320 16177072

[58] Bsibsi M Persoon-Deen C Verwer RW Meeuwsen S Ravid R Van Noort JM. Toll-like receptor 3 on adult human astrocytes triggers production of neuroprotective mediators. Glia (2006) 53:688–95. 10.1002/glia.20328 16482523

[59] Weber F Meinl E Aloisi F Nevinny-Stickel C Albert E Wekerle H Human astrocytes are only partially competent antigen presenting cells. Possible implications for lesion development in multiple sclerosis. Brain (1994) 117:59–69. 10.1093/brain/117.1.59 7511974

[60] Cornet A Bettelli E Oukka M Cambouris C Avellana-Adalid V Kosmatopoulos K Role of astrocytes in antigen presentation and naive T-cell activation. J Neuroimmunol (2000) 106:69–77. 10.1016/S0165-5728(99)00215-5 10814784

[61] Michel L Touil H Pikor NB Gommerman JL Prat A Bar-Or A. B cells in the multiple sclerosis central nervous system: trafficking and contribution to CNS-compartmentalized inflammation. Front Immunol (2015) 6:636. 10.3389/fimmu.2015.00636 26732544 PMC4689808

[62] Khan WN. B cell receptor and BAFF receptor signaling regulation of B cell homeostasis. J Immunol (2009) 183:3561–7. 10.4049/jimmunol.0800933 19726767

[63] Krumbholz M Theil D Derfuss T Rosenwald A Schrader F Monoranu C-M BAFF is produced by astrocytes and up-regulated in multiple sclerosis lesions and primary central nervous system lymphoma. J Exp Med (2005) 201:195–200. 10.1084/jem.20041674 15642740 PMC2212784

[64] Gavillet M Allaman I Magistretti PJ. Modulation of astrocytic metabolic phenotype by proinflammatory cytokines. Glia (2008) 56:975–89. 10.1002/glia.20671 18383346

[65] Fang J Han D Hong J Tan Q Tian Y. The chemokine, macrophage inflammatory protein-2γ, reduces the expression of glutamate transporter-1 on astrocytes and increases neuronal sensitivity to glutamate excitotoxicity. J Neuroinflammtion (2012) 9:267–267. 10.1186/1742-2094-9-267 23234294 PMC3545864

[66] Werner P Pitt D Raine CS. Multiple sclerosis: altered glutamate homeostasis in lesions correlates with oligodendrocyte and axonal damage. Ann Neurol (2001) 50:169–80. 10.1002/ana.1077 11506399

[67] Liddelow SA Barres BA. Reactive astrocytes: production, function, and therapeutic potential. Immunity (2017) 46:957–67. 10.1016/j.immuni.2017.06.006 28636962

[68] Liddelow SA Guttenplan KA Clarke LE Bennett FC Bohlen CJ Schirmer L Neurotoxic reactive astrocytes are induced by activated microglia. Nature (2017) 541:481–7. 10.1038/nature21029 28099414 PMC5404890

[69] Martinez FO Gordon S. The M1 and M2 paradigm of macrophage activation: time for reassessment. F1000Prime Rep (2014) 6:13. 10.12703/P6-13 24669294 PMC3944738

[70] Pekny M Pekna M Messing A Steinhäuser C Lee J-M Parpura V Astrocytes: a central element in neurological diseases. Acta Neuropathol (2016) 131:323–45. 10.1007/s00401-015-1513-1 26671410

[71] Zamanian JL Xu L Foo LC Nouri N Zhou L Giffard RG Genomic analysis of reactive astrogliosis. J Neurosci (2012) 32:6391–410. 10.1523/JNEUROSCI.6221-11.2012 22553043 PMC3480225

[72] Cordiglieri C Farina C Astrocytes exert and control immune responses in the brain. Curr Immunol Rev (2010) 6:150 10.2174/157339510791823655

[73] Hulshof S Montagne L De Groot CJ Van Der Valk P. Cellular localization and expression patterns of interleukin-10, interleukin-4, and their receptors in multiple sclerosis lesions. Glia (2002) 38:24–35. 10.1002/glia.10050 11921201

[74] Wang D Ayers MM Catmull DV Hazelwood LJ Bernard CC Orian JM. Astrocyte-associated axonal damage in pre-onset stages of experimental autoimmune encephalomyelitis. Glia (2005) 51:235–40. 10.1002/glia.20199 15812814

[75] Pham H Ramp AA Klonis N Ng SW Klopstein A Ayers MM The astrocytic response in early experimental autoimmune encephalomyelitis occurs across both the grey and white matter compartments. J Neuroimmunol (2009) 208:30–9. 10.1016/j.jneuroim.2008.12.010 19195719

[76] D’Amelio FE Smith ME Eng LF. Sequence of tissue responses in the early stages of experimental allergic encephalomyelitis (EAE): immunohistochemical, light microscopic, and ultrastructural observations in the spinal cord. Glia (1990) 3:229–40. 10.1002/glia.440030402 2144503

[77] Zeinstra E Wilczak N De Keyser J. Reactive astrocytes in chronic active lesions of multiple sclerosis express co-stimulatory molecules B7-1 and B7-2. J Neuroimmunol (2003) 135:166–71. 10.1016/S0165-5728(02)00462-9 12576238

[78] Traugott U. Multiple sclerosis: relevance of class I and class II MHC-expressing cells to lesion development. J Neuroimmunol (1987) 16:283–302. 10.1016/0165-5728(87)90082-8 3114324

[79] Meinl E Aloisi F Ertl B Weber F de Waal Malefyt R Wekerle H Multiple sclerosis. Immunomodulatory effects of human astrocytes on T cells. Brain (1994) 117(Pt 6):1323–32. 10.1093/brain/117.6.1323 7820569

[80] Sørensen TL Tani M Jensen J Pierce V Lucchinetti C Folcik VA Expression of specific chemokines and chemokine receptors in the central nervous system of multiple sclerosis patients. J Clin Invest (1999) 103:807–15. 10.1172/JCI5150 10079101 PMC408141

[81] Ponath G Lincoln MR Dahlawi S Mubarak M Sumida T Airas L Enhanced astrocyte responses are driven by a genetic risk allele associated with multiple sclerosis. bioRxiv (2017). 10.1101/206110 PMC6297228 30559390

[82] Love S Louis D Ellison DW Sobel RA Moore GRW Chapter 20: Demyelinating diseases. Greenfield’s Neuropathology. (Vol. 2) 8th edn CRC Press (2008). p. 1513–608.

[83] Kim RY Hoffman AS Itoh N Ao Y Spence R Sofroniew MV Astrocyte CCL2 sustains immune cell infiltration in chronic experimental autoimmune encephalomyelitis. J Neuroimmunol (2014) 274:53–61. 10.1016/j.jneuroim.2014.06.009 25005117 PMC4343306

[84] Bullard DC Hu X Schoeb TR Collins RG Beaudet AL Barnum SR. Intercellular adhesion molecule-1 expression is required on multiple cell types for the development of experimental autoimmune encephalomyelitis. J Immunol (2007) 178:851–7. 10.4049/jimmunol.178.2.851 17202346

[85] Kipp M Clarner T Dang J Copray S Beyer C. The cuprizone animal model: new insights into an old story. Acta Neuropathol (2009) 118:723–36. 10.1007/s00401-009-0591-3 19763593

[86] Skripuletz T Hackstette D Bauer K Gudi V Pul R Voss E Astrocytes regulate myelin clearance through recruitment of microglia during cuprizone-induced demyelination. Brain (2013) 136:147–67. 10.1093/brain/aws262 23266461

[87] Ragheb S Li Y Simon K VanHaerents S Galimberti D De Riz M Multiple sclerosis: BAFF and CXCL13 in cerebrospinal fluid. Mult Scler (2011) 17:819–29. 10.1177/1352458511398887 21372118

[88] Stern JN Yaari G Vander Heiden JA Church G Donahue WF Hintzen RQ B cells populating the multiple sclerosis brain mature in the draining cervical lymph nodes. Sci Transl Med (2014) 6:248ra107. 10.1126/scitranslmed.3008879 25100741 PMC4388137

[89] Magliozzi R Howell O Vora A Serafini B Nicholas R Puopolo M Meningeal B-cell follicles in secondary progressive multiple sclerosis associate with early onset of disease and severe cortical pathology. Brain (2007) 130:1089–104. 10.1093/brain/awm038 17438020

[90] Srinivasan R Sailasuta N Hurd R Nelson S Pelletier D. Evidence of elevated glutamate in multiple sclerosis using magnetic resonance spectroscopy at 3 T. Brain (2005) 128:1016–25. 10.1093/brain/awh467 15758036

[91] Baranzini SE Srinivasan R Khankhanian P Okuda DT Nelson SJ Matthews PM Genetic variation influences glutamate concentrations in brains of patients with multiple sclerosis. Brain (2010) 133:2603–11. 10.1093/brain/awq192 20802204 PMC2929334

[92] Won JS Singh AK Singh I. Lactosylceramide: a lipid second messenger in neuroinflammatory disease. J Neurochem (2007) 1(103 Suppl):180–91. 10.1111/j.1471-4159.2007.04822.x 17986153

[93] Huang EJ Reichardt LF. Neurotrophins: roles in neuronal development and function. Annu Rev Neurosci (2001) 24:677–736. 10.1146/annurev.neuro.24.1.677 11520916 PMC2758233

[94] Lee D-H Geyer E Flach A-C Jung K Gold R Flügel A Central nervous system rather than immune cell-derived BDNF mediates axonal protective effects early in autoimmune demyelination. Acta Neuropathol (2012) 123:247–58. 10.1007/s00401-011-0890-3 22009304 PMC3259380

[95] Linker RA Lee DH Demir S Wiese S Kruse N Siglienti I Functional role of brain-derived neurotrophic factor in neuroprotective autoimmunity: therapeutic implications in a model of multiple sclerosis. Brain (2010) 133:2248–63. 10.1093/brain/awq179 20826430

[96] Fulmer CG VonDran MW Stillman AA Huang Y Hempstead BL Dreyfus CF. Astrocyte-derived BDNF supports myelin protein synthesis after cuprizone-induced demyelination. J Neurosci (2014) 34:8186–96. 10.1523/JNEUROSCI.4267-13.2014 24920623 PMC4051974

[97] Colombo E Cordiglieri C Melli G Newcombe J Krumbholz M Parada LF Stimulation of the neurotrophin receptor TrkB on astrocytes drives nitric oxide production and neurodegeneration. J Exp Med (2012) 209:521. 10.1084/jem.20110698 22393127 PMC3302220

[98] Stadelmann C Kerschensteiner M Misgeld T Brück W Hohlfeld R Lassmann H. BDNF and gp145trkB in multiple sclerosis brain lesions: neuroprotective interactions between immune and neuronal cells? Brain (2002) 125:75–85. 10.1093/brain/awf015 11834594

[99] Dodel RC Du Y Bales KR Gao F Paul SM. Sodium salicylate and 17beta-estradiol attenuate nuclear transcription factor NF-kappaB translocation in cultured rat astroglial cultures following exposure to amyloid A beta(1-40) and lipopolysaccharides. J Neurochem (1999) 73:1453–60. 10.1046/j.1471-4159.1999.0731453.x 10501189

[100] Tenenbaum M Azab AN Kaplanski J. Effects of estrogen against LPS-induced inflammation and toxicity in primary rat glial and neuronal cultures. J Endotoxin Res (2007) 13:158–66. 10.1177/0968051907080428 17621558

[101] Cerciat M Unkila M Garcia-Segura LM Arevalo MA. Selective estrogen receptor modulators decrease the production of interleukin-6 and interferon-gamma-inducible protein-10 by astrocytes exposed to inflammatory challenge in vitro. Glia (2010) 58:93–102. 10.1002/glia.20904 19533603

[102] Spence RD Hamby ME Umeda E Itoh N Du S Wisdom AJ Neuroprotection mediated through estrogen receptor-α in astrocytes. Proc Natl Acad Sci U S A (2011) 108:8867–72. 10.1073/pnas.1103833108 21555578 PMC3102368

[103] Spence RD Wisdom AJ Cao Y Hill HM Mongerson CRL Stapornkul B estrogen mediates neuroprotection and anti-inflammatory effects during EAE through ERα signaling on astrocytes but not through ERβ signaling on astrocytes or neurons. J Neurosci (2013) 33:10924–33. 10.1523/JNEUROSCI.0886-13.2013 23804112 PMC3693061

[104] Luchetti S van Eden CG Schuurman K van Strien ME Swaab DF Huitinga I. Gender differences in multiple sclerosis: induction of estrogen signaling in male and progesterone signaling in female lesions. J Neuropathol Exp Neurol (2014) 73:123–35. 10.1097/NEN.0000000000000037 24423637

[105] Noorbakhsh F Ellestad KK Maingat F Warren KG Han MH Steinman L Impaired neurosteroid synthesis in multiple sclerosis. Brain (2011) 134:2703–21. 10.1093/brain/awr200 21908875 PMC4141444

[106] Touil T Fitzgerald D Zhang GX Rostami A Gran B. Cutting edge: TLR3 stimulation suppresses experimental autoimmune encephalomyelitis by inducing endogenous IFN-beta. J Immunol (2006) 177:7505–9. 10.4049/jimmunol.177.11.7505 17114417

[107] Marta M Andersson A Isaksson M Kampe O Lobell A. Unexpected regulatory roles of TLR4 and TLR9 in experimental autoimmune encephalomyelitis. Eur J Immunol (2008) 38:565–75. 10.1002/eji.200737187 18203139

[108] Berer K Mues M Koutrolos M Rasbi ZA Boziki M Johner C Commensal microbiota and myelin autoantigen cooperate to trigger autoimmune demyelination. Nature (2011) 479:538–41. 10.1038/nature10554 22031325

[109] Jangi S Gandhi R Cox LM Li N von Glehn F Yan R Alterations of the human gut microbiome in multiple sclerosis. Nat Commun (2016) 7:12015. 10.1038/ncomms12015 27352007 PMC4931233

[110] Braniste V Al-Asmakh M Kowal C Anuar F Abbaspour A Toth M The gut microbiota influences blood-brain barrier permeability in mice. Sci Transl Med (2014) 6:263ra158. 10.1126/scitranslmed.3009759 25411471 PMC4396848

[111] Fessler EB Chibane FL Wang Z Chuang DM. Potential roles of HDAC inhibitors in mitigating ischemia-induced brain damage and facilitating endogenous regeneration and recovery. Curr Pharm Des (2013) 19:5105–20. 10.2174/1381612811319280009 23448466 PMC6322545

[112] Michel L Prat A One more role for the gut: microbiota and blood brain barrier. Ann Transl Med (2016) 4:15 10.3978/j.issn.2305-5839.2015.10.16 26855951 PMC4716932

[113] Shih R-H Wang C-Y Yang C-M. NF-kappaB signaling pathways in neurological inflammation: a mini review. Front Mol Neurosci (2015) 8:77. 10.3389/fnmol.2015.00077 26733801 PMC4683208

[114] Hayden MS Ghosh S NF-kappaB, the first quarter-century: remarkable progress and outstanding questions. Genes Dev (2012) 26:203–34. 10.1101/gad.183434.111 22302935 PMC3278889

[115] Mc Guire C Prinz M Beyaert R van Loo G Nuclear factor kappa B (NF-κB) in multiple sclerosis pathology. Trends Mol Med (2013) 19:604–13. 10.1016/j.molmed.2013.08.001 24007818

[116] Kawai T Akira S Signaling to NF-κB by Toll-like receptors. Trends Mol Med (2007) 13:460–9. 10.1016/j.molmed.2007.09.002 18029230

[117] Brambilla R Persaud T Hu X Karmally S Shestopalov VI Dvoriantchikova G Transgenic inhibition of astroglial NF-κB improves functional outcome in experimental autoimmune encephalomyelitis by suppressing chronic central nervous system inflammation. J Immunol (2009) 182:2628–40. 10.4049/jimmunol.0802954 19234157 PMC4291126

[118] Brambilla R Bracchi-Ricard V Hu WH Frydel B Bramwell A Karmally S Inhibition of astroglial nuclear factor kappaB reduces inflammation and improves functional recovery after spinal cord injury. J Exp Med (2005) 202:145–56. 10.1084/jem.20041918 15998793 PMC2212896

[119] Dvoriantchikova G Barakat D Brambilla R Agudelo C Hernandez E Bethea JR Inactivation of astroglial NF-kappa B promotes survival of retinal neurons following ischemic injury. Eur J Neurosci (2009) 30:175–85. 10.1111/j.1460-9568.2009.06814.x 19614983 PMC2778328

[120] Kang Z Wang C Zepp J Wu L Sun K Zhao J Act1 mediates IL-17-induced EAE pathogenesis selectively in NG2(+) glial cells. Nat Neurosci (2013) 16:1401–8. 10.1038/nn.3505 23995070 PMC4106025

[121] Rothhammer V Mascanfroni ID Bunse L Takenaka MC Kenison JE Mayo L Type I interferons and microbial metabolites of tryptophan modulate astrocyte activity and central nervous system inflammation via the aryl hydrocarbon receptor. Nat Med (2016) 22:586–97. 10.1038/nm.4106 27158906 PMC4899206

[122] Vogel CF Matsumura F. A new cross-talk between the aryl hydrocarbon receptor and RelB, a member of the NF-kappaB family. Biochem Pharmacol (2009) 77:734–45. 10.1016/j.bcp.2008.09.036 18955032 PMC2688397

[123] Housley WJ Fernandez SD Vera K Murikinati SR Grutzendler J Cuerdon N Genetic variants associated with autoimmunity drive NFκB signaling and responses to inflammatory stimuli. Sci Transl Med (2015) 7:ra93–291. 10.1126/scitranslmed.aaa9223 26062845 PMC4574294

[124] Nicolas CS Amici M Bortolotto ZA Doherty A Csaba Z Fafouri A The role of JAK-STAT signaling within the CNS. JAKSTAT (2013) 2:e22925. 10.4161/jkst.22925 24058789 PMC3670265

[125] Ernst M Jenkins BJ. Acquiring signalling specificity from the cytokine receptor gp130. Trends Genet (2004) 20:23–32. 10.1016/j.tig.2003.11.003 14698616

[126] Park EJ Ji KA Jeon SB Choi WH Han IO You HJ Rac1 contributes to maximal activation of STAT1 and STAT3 in IFN-gamma-stimulated rat astrocytes. J Immunol (2004) 173:5697–703. 10.4049/jimmunol.173.9.5697 15494521

[127] Choi WH Ji KA Jeon SB Yang MS Kim H Min KJ Anti-inflammatory roles of retinoic acid in rat brain astrocytes: suppression of interferon-gamma-induced JAK/STAT phosphorylation. Biochem Biophys Res Commun (2005) 329:125–31. 10.1016/j.bbrc.2005.01.110 15721283

[128] Herrmann JE Imura T Song B Qi J Ao Y Nguyen TK STAT3 is a critical regulator of astrogliosis and scar formation after spinal cord injury. J Neurosci (2008) 28:7231–43. 10.1523/JNEUROSCI.1709-08.2008 18614693 PMC2583788

[129] Okada S Nakamura M Katoh H Miyao T Shimazaki T Ishii K Conditional ablation of Stat3 or Socs3 discloses a dual role for reactive astrocytes after spinal cord injury. Nat Med (2006) 12:829–34. 10.1038/nm1425 16783372

[130] Haroon F Drogemuller K Handel U Brunn A Reinhold D Nishanth G Gp130-dependent astrocytic survival is critical for the control of autoimmune central nervous system inflammation. J Immunol (2011) 186:6521–31. 10.4049/jimmunol.1001135 21515788

[131] Sticozzi C Belmonte G Meini A Carbotti P Grasso G Palmi M IL-1beta induces GFAP expression in vitro and in vivo and protects neurons from traumatic injury-associated apoptosis in rat brain striatum via NFkappaB/Ca(2)(+)-calmodulin/ERK mitogen-activated protein kinase signaling pathway. Neuroscience (2013) 252:367–83. 10.1016/j.neuroscience.2013.07.061 23928073

[132] Kim JA Yun H-M Jin P Lee HP Han JY Udumula V Inhibitory effect of a 2,4-bis(4-hydroxyphenyl)-2-butenal diacetate on neuro-inflammatory reactions via inhibition of STAT1 and STAT3 activation in cultured astrocytes and microglial BV-2 cells. Neuropharmacology (2014) 79:476–87. 10.1016/j.neuropharm.2013.06.032 23891616

[133] Sun L Li Y Jia X Wang Q Li Y Hu M Neuroprotection by IFN-γ via astrocyte-secreted IL-6 in acute neuroinflammation. Oncotarget (2017) 8:40065–78. 10.18632/oncotarget.16990 28454116 PMC5522245

[134] Kimura A Kishimoto T. IL-6: regulator of TREG/Th17 balance. Eur J Immunol (2010) 40:1830–5. 10.1002/eji.201040391 20583029

[135] Erta M Quintana A Hidalgo J. Interleukin-6, a major cytokine in the central nervous system. Int J Biol Sci (2012) 8:1254–66. 10.7150/ijbs.4679 23136554 PMC3491449

[136] Comi G Jeffery D Kappos L Montalban X Boyko A Rocca MA Placebo-controlled trial of oral laquinimod for multiple sclerosis. N Engl J Med (2012) 366:1000–9. 10.1056/NEJMoa1104318 22417253

[137] Barkhof F Giovannoni G Hartung H-P Cree B Uccelli A Sormani MP ARPEGGIO: a randomized, placebo-controlled study to evaluate oral laquinimod in patients with primary progressive multiple sclerosis (PPMS) (P7.210). Neurology (2015) 84:P7.210.

[138] Kaye J Piryatinsky V Birnberg T Hingaly T Raymond E Kashi R Laquinimod arrests experimental autoimmune encephalomyelitis by activating the aryl hydrocarbon receptor. Proc Natl Acad Sci U S A (2016) 113:E6145–52. 10.1073/pnas.1607843113 27671624 PMC5068259

[139] Abel J Haarmann-Stemmann T. An introduction to the molecular basics of aryl hydrocarbon receptor biology. Biol Chem (2010) 391:1235–48. 10.1515/BC.2010.128 20868221

[140] Denison MS Nagy SR. Activation of the aryl hydrocarbon receptor by structurally diverse exogenous and endogenous chemicals. Annu Rev Pharmacol Toxicol (2003) 43:309–34. 10.1146/annurev.pharmtox.43.100901.135828 12540743

[141] Fritsche E Schafer C Calles C Bernsmann T Bernshausen T Wurm M Lightening up the UV response by identification of the arylhydrocarbon receptor as a cytoplasmatic target for ultraviolet B radiation. Proc Natl Acad Sci U S A (2007) 104:8851–6. 10.1073/pnas.0701764104 17502624 PMC1885591

[142] Madhukar BV Brewster DW Matsumura F. Effects of in vivo-administered 2,3,7,8-tetrachlorodibenzo-p-dioxin on receptor binding of epidermal growth factor in the hepatic plasma membrane of rat, guinea pig, mouse, and hamster. Proc Natl Acad Sci U S A (1984) 81:7407–11. 10.1073/pnas.81.23.7407 6095293 PMC392155

[143] Oesch-Bartlomowicz B Huelster A Wiss O Antoniou-Lipfert P Dietrich C Arand M Aryl hydrocarbon receptor activation by cAMP vs. dioxin: divergent signaling pathways. Proc Natl Acad Sci U S A (2005) 102:9218–23. 10.1073/pnas.0503488102 15972329 PMC1154791

[144] Vogel CF Sciullo E Li W Wong P Lazennec G Matsumura F. RelB, a new partner of aryl hydrocarbon receptor-mediated transcription. Mol Endocrinol (2007) 21:2941–55. 10.1210/me.2007-0211 17823304 PMC2346533

[145] Tian Y Rabson AB Gallo MA. Ah receptor and NF-kappaB interactions: mechanisms and physiological implications. Chem Biol Interact (2002) 141:97–115. 10.1016/S0009-2797(02)00068-6 12213387

[146] Vogel CFA Haarmann-Stemmann T The aryl hydrocarbon receptor repressor – More than a simple feedback inhibitor of AhR signaling: clues for its role in inflammation and cancer. Curr Opin Toxicol (2017) 2:109–19. 10.1016/j.cotox.2017.02.004 28971163 PMC5621755

[147] Wegner C Stadelmann C Pfortner R Raymond E Feigelson S Alon R Laquinimod interferes with migratory capacity of T cells and reduces IL-17 levels, inflammatory demyelination and acute axonal damage in mice with experimental autoimmune encephalomyelitis. J Neuroimmunol (2010) 227:133–43. 10.1016/j.jneuroim.2010.07.009 20684995

[148] Yang JS Xu LY Xiao BG Hedlund G Link H. Laquinimod (ABR-215062) suppresses the development of experimental autoimmune encephalomyelitis, modulates the Th1/Th2 balance and induces the Th3 cytokine TGF-beta in Lewis rats. J Neuroimmunol (2004) 156:3–9. 10.1016/j.jneuroim.2004.02.016 15465591

[149] Bruck W Pfortner R Pham T Zhang J Hayardeny L Piryatinsky V Reduced astrocytic NF-kappaB activation by laquinimod protects from cuprizone-induced demyelination. Acta Neuropathol (2012) 124:411–24. 10.1007/s00401-012-1009-1 22766690 PMC3422618

[150] Thone J Ellrichmann G Seubert S Peruga I Lee DH Conrad R Modulation of autoimmune demyelination by laquinimod via induction of brain-derived neurotrophic factor. Am J Pathol (2012) 180:267–74. 10.1016/j.ajpath.2011.09.037 22152994

[151] Fox RJ Miller DH Phillips JT Hutchinson M Havrdova E Kita M Placebo-controlled phase 3 study of oral BG-12 or glatiramer in multiple sclerosis. N Engl J Med (2012) 367:1087–97. 10.1056/NEJMoa1206328 22992072

[152] Gold R Kappos L Arnold DL Bar-Or A Giovannoni G Selmaj K Placebo-controlled phase 3 study of oral BG-12 for relapsing multiple sclerosis. N Engl J Med (2012) 367:1098–107. 10.1056/NEJMoa1114287 22992073

[153] Biogen axes 11% of Workforce in Restructuring. New Rochelle, NY: Genetic Engineering & Biotechnology News (2015). Available from: http://www.genengnews.com/gen-news-highlights/biogen-axes-11-of-workforce-in-restructuring/81251879

[154] Scannevin RH Chollate S Jung MY Shackett M Patel H Bista P Fumarates promote cytoprotection of central nervous system cells against oxidative stress via the nuclear factor (Erythroid-Derived 2)-like 2 pathway. J Pharmacol Exp Ther (2012) 341:274. 10.1124/jpet.111.190132 22267202

[155] Lehmann JCU Listopad JJ Rentzsch CU Igney FH von Bonin A Hennekes HH Dimethylfumarate induces immunosuppression via glutathione depletion and subsequent induction of heme oxygenase 1. J Invest Dermatol (2007) 127:835–45. 10.1038/sj.jid.5700686 17235328

[156] Gold R Linker RA Stangel M. Fumaric acid and its esters: an emerging treatment for multiple sclerosis with antioxidative mechanism of action. Clin Immunol (2012) 142:44–8. 10.1016/j.clim.2011.02.017 21414846

[157] Brennan MS Matos MF Li B Hronowski X Gao B Juhasz P Dimethyl fumarate and monoethyl fumarate exhibit differential effects on KEAP1, NRF2 activation, and glutathione depletion in vitro. PLoS One (2015) 10:e0120254. 10.1371/journal.pone.0120254 25793262 PMC4368598

[158] Itoh K Wakabayashi N Katoh Y Ishii T Igarashi K Engel JD Keap1 represses nuclear activation of antioxidant responsive elements by Nrf2 through binding to the amino-terminal Neh2 domain. Genes Dev (1999) 13:76–86. 10.1101/gad.13.1.76 9887101 PMC316370

[159] Gillard GO Collette B Anderson J Chao J Scannevin RH Huss DJ DMF, but not other fumarates, inhibits NF-κB activity in vitro in an Nrf2-independent manner. J Neuroimmunol (2015) 283:74–85. 10.1016/j.jneuroim.2015.04.006 26004161

[160] Blewett MM Xie J Zaro BW Backus KM Altman A Teijaro JR Chemical proteomic map of dimethyl fumarate–sensitive cysteines in primary human T cells. Sci Signal (2016) 9:rs10–10. 10.1126/scisignal.aaf7694 27625306 PMC5068918

[161] Diebold M Sievers C Bantug G Sanderson N Kappos L Kuhle J Dimethyl fumarate influences innate and adaptive immunity in multiple sclerosis. J Autoimmun (2017) 86:39–50. 10.1016/j.jaut.2017.09.009 28958667

[162] Brennan MS Matos MF Richter KE Li B Scannevin RH. The NRF2 transcriptional target, OSGIN1, contributes to monomethyl fumarate-mediated cytoprotection in human astrocytes. Sci Rep (2017) 7:42054. 10.1038/srep42054 28181536 PMC5299414

[163] Magliozzi R Howell OW Reeves C Roncaroli F Nicholas R Serafini B of neuronal loss and meningeal inflammation in multiple sclerosis. Ann Neurol (2010) 68:477–93. 10.1002/ana.22230 20976767

[164] Galloway DA Williams JB Moore CS. Effects of fumarates on inflammatory human astrocyte responses and oligodendrocyte differentiation. Ann Clin Transl Neurol (2017) 4:381–91. 10.1002/acn3.414 28589165 PMC5454401

[165] Kappos L Radue EW O’Connor P Polman C Hohlfeld R Calabresi P A placebo-controlled trial of oral fingolimod in relapsing multiple sclerosis. N Engl J Med (2010) 362:387–401. 10.1056/NEJMoa0909494 20089952

[166] Calabresi PA Radue EW Goodin D Jeffery D Rammohan KW Reder AT Safety and efficacy of fingolimod in patients with relapsing-remitting multiple sclerosis (FREEDOMS II): a double-blind, randomised, placebo-controlled, phase 3 trial. Lancet Neurol (2014) 13:545–56. 10.1016/S1474-4422(14)70049-3 24685276

[167] Smith AL Cohen JA Multiple sclerosis: fingolimod failure in progressive MS INFORMS future trials. Nat Rev Neurol (2016) 12:253–4. 10.1038/nrneurol.2016.37 27020561

[168] Fox RJ Effects of siponimod on MRI outcomes in patients with secondary progressive multiple sclerosis: results of the phase 3 EXPAND study. ECTRIMS Online Libr (2017) 26:202482.

[169] Matloubian M Lo CG Cinamon G Lesneski MJ Xu Y Brinkmann V Lymphocyte egress from thymus and peripheral lymphoid organs is dependent on S1P receptor 1. Nature (2004) 427:355–60. 10.1038/nature02284 14737169

[170] Pham TH Okada T Matloubian M Lo CG Cyster JG. S1P1 receptor signaling overrides retention mediated by G alpha i-coupled receptors to promote T cell egress. Immunity (2008) 28:122–33. 10.1016/j.immuni.2007.11.017 18164221 PMC2691390

[171] Pebay A Toutant M Premont J Calvo CF Venance L Cordier J Sphingosine-1-phosphate induces proliferation of astrocytes: regulation by intracellular signalling cascades. Eur J Neurosci (2001) 13:2067–76. 10.1046/j.0953-816x.2001.01585.x 11467306

[172] Mullershausen F Craveiro LM Shin Y Cortes-Cros M Bassilana F Osinde M Phosphorylated FTY720 promotes astrocyte migration through sphingosine-1-phosphate receptors. J Neurochem (2007) 102:1151–61. 10.1111/j.1471-4159.2007.04629.x 17488279

[173] Jaillard C Harrison S Stankoff B Aigrot MS Calver AR Duddy G Edg8/S1P5: an oligodendroglial receptor with dual function on process retraction and cell survival. J Neurosci (2005) 25:1459–69. 10.1523/JNEUROSCI.4645-04.2005 15703400 PMC6726002

[174] Saini HS Coelho RP Goparaju SK Jolly PS Maceyka M Spiegel S Novel role of sphingosine kinase 1 as a mediator of neurotrophin-3 action in oligodendrocyte progenitors. J Neurochem (2005) 95:1298–310. 10.1111/j.1471-4159.2005.03451.x 16313513

[175] MacLennan AJ Devlin BK Marks L Gaskin AA Neitzel KL Lee N. Antisense studies in PC12 cells suggest a role for H218, a sphingosine 1-phosphate receptor, in growth-factor-induced cell-cell interaction and neurite outgrowth. Dev Neurosci (2000) 22:283–95. 10.1159/000017452 10965150

[176] Harada J Foley M Moskowitz MA Waeber C. Sphingosine-1-phosphate induces proliferation and morphological changes of neural progenitor cells. J Neurochem (2004) 88:1026–39. 10.1046/j.1471-4159.2003.02219.x 14756825

[177] Sato K Tomura H Igarashi Y Ui M Okajima F. Exogenous sphingosine 1-phosphate induces neurite retraction possibly through a cell surface receptor in PC12 cells. Biochem Biophys Res Commun (1997) 240:329–34. 10.1006/bbrc.1997.7666 9388477

[178] Choi JW Gardell SE Herr DR Rivera R Lee C-W Noguchi K FTY720 (fingolimod) efficacy in an animal model of multiple sclerosis requires astrocyte sphingosine 1-phosphate receptor 1 (S1P1) modulation. Proc Natl Acad Sci U S A (2011) 108:751–6. 10.1073/pnas.1014154108 21177428 PMC3021041

[179] Malchinkhuu E Sato K Muraki T Ishikawa K Kuwabara A Okajima F. Assessment of the role of sphingosine 1-phosphate and its receptors in high-density lipoprotein-induced stimulation of astroglial cell function. Biochem J (2003) 370:817–27. 10.1042/bj20020867 12470300 PMC1223227

[180] Van Doorn R Van Horssen J Verzijl D Witte M Ronken E Van Het Hof B Sphingosine 1-phosphate receptor 1 and 3 are upregulated in multiple sclerosis lesions. Glia (2010) 58:1465–76. 10.1002/glia.21021 20648639

[181] Colombo E Di Dario M Capitolo E Chaabane L Newcombe J Martino G Fingolimod may support neuroprotection via blockade of astrocyte nitric oxide. Ann Neurol (2014) 76:325–37. 10.1002/ana.24217 25043204

[182] Hoffmann FS Hofereiter J Rubsamen H Melms J Schwarz S Faber H Fingolimod induces neuroprotective factors in human astrocytes. J Neuroinflammation (2015) 12:184. 10.1186/s12974-015-0393-6 26419927 PMC4589103

[183] Wu C Leong SY Moore CS Cui QL Gris P Bernier L-P Dual effects of daily FTY720 on human astrocytes in vitro: relevance for neuroinflammation. J Neuroinflammation (2013) 10:41–41. 10.1186/1742-2094-10-41 23509960 PMC3621211

[184] Cohen JA Chun J. Mechanisms of fingolimod’s efficacy and adverse effects in multiple sclerosis. Ann Neurol (2011) 69:759–77. 10.1002/ana.22426 21520239

[185] Edling A Woodworth L Agrawal R Mahan A Garron T Hagan N Teriflunomide impacts primary microglia and astrocyte functions in vitro (P2.348). Neurology (2017) 88:P2.348.

[186] Miljkovic D Samardzic T Mostarica Stojkovic M Stosic-Grujicic S Popadic D Trajkovic V. Leflunomide inhibits activation of inducible nitric oxide synthase in rat astrocytes. Brain Res (2001) 889:331–8. 10.1016/S0006-8993(00)03181-4 11166726

[187] Limsakun T Menguy-Vacheron F Pharmacokinetics of oral teriflunomide, a novel oral disease-modifying agent under investigation for the treatment of multiple sclerosis. Neurology (2010) 74:A415.

[188] Tallantyre E Evangelou N Constantinescu CS. Spotlight on teriflunomide. Int MS J (2008) 15:62–8. 18782502

